# The Pharmacological Activity, Biochemical Properties, and Pharmacokinetics of the Major Natural Polyphenolic Flavonoid: Quercetin

**DOI:** 10.3390/foods9030374

**Published:** 2020-03-23

**Authors:** Gaber El-Saber Batiha, Amany Magdy Beshbishy, Muhammad Ikram, Zohair S. Mulla, Mohamed E. Abd El-Hack, Ayman E. Taha, Abdelazeem M. Algammal, Yaser Hosny Ali Elewa

**Affiliations:** 1 National Research Center for Protozoan Diseases, Obihiro University of Agriculture and Veterinary Medicine, Nishi 2-13, Inada-cho, Obihiro 080-8555, Japan; amanimagdi2008@gmail.com; 2 Department of Pharmacology and Therapeutics, Faculty of Veterinary Medicine, Damanhour University, Damanhour 22511, Egypt; 3 Department of Chemistry, Abdul Wali Khan University Mardan, Mardan 23200, Pakistan; ikrambiochem2014@gmail.com; 4 Department of Public Health, College of Veterinary Medicine, King Faisal University, Hofuf 31982, Saudi Arabia; drzomu@gmail.com; 5 Department of Poultry, Faculty of Agriculture, Zagazig University, Zagazig 44511, Egypt; dr.mohamed.e.abdalhaq@gmail.com; 6 Department of Animal Husbandry and Animal Wealth Development, Faculty of Veterinary Medicine, Alexandria University, Edfina 22758, Egypt; Ayman.Taha@alexu.edu.eg; 7 Department of Bacteriology, Immunology and Mycology, Faculty of Veterinary Medicine, Suez Canal University, Ismailia 41522, Egypt; abdelazeem.algammal@vet.suez.edu.eg or; 8 Department of Histology and Cytology, Faculty of Veterinary Medicine, Zagazig University, Zagazig 44519, Egypt; y-elewa@vetmed.hokudai.ac.jp; 9 Laboratory of Anatomy, Department of Biomedical Sciences, Graduate School of Veterinary Medicine, Hokkaido University, Sapporo 060-0818, Japan

**Keywords:** quercetin, herbal remedies, pharmacological activities, pharmacokinetics, Alzheimer’s disease

## Abstract

Flavonoids are a class of natural substances present in plants, fruits, vegetables, wine, bulbs, bark, stems, roots, and tea. Several attempts are being made to isolate such natural products, which are popular for their health benefits. Flavonoids are now seen as an essential component in a number of cosmetic, pharmaceutical, and medicinal formulations. Quercetin is the major polyphenolic flavonoid found in food products, including berries, apples, cauliflower, tea, cabbage, nuts, and onions that have traditionally been treated as anticancer and antiviral, and used for the treatment of allergic, metabolic, and inflammatory disorders, eye and cardiovascular diseases, and arthritis. Pharmacologically, quercetin has been examined against various microorganisms and parasites, including pathogenic bacteria, viruses, and *Plasmodium*, *Babesia,* and *Theileria* parasites. Additionally, it has shown beneficial effects against Alzheimer’s disease (AD), and this activity is due to its inhibitory effect against acetylcholinesterase. It has also been documented to possess antioxidant, antifungal, anti-carcinogenic, hepatoprotective, and cytotoxic activity. Quercetin has been documented to accumulate in the lungs, liver, kidneys, and small intestines, with lower levels seen in the brain, heart, and spleen, and it is extracted through the renal, fecal, and respiratory systems. The current review examines the pharmacokinetics, as well as the toxic and biological activities of quercetin.

## 1. Introduction

Plants have been used since ancient times to cure certain infectious diseases, some of which are now standard treatments for several diseases [1,2]. Over the last decade, there has been a huge increase in acceptance and public interest in natural therapy in both developing and developed countries, and these herbal medicines are now available, not only in drug stores but also in supermarkets and food stores. Approximately 80 percent of people in Africa and other developing nations still depend on traditional herbal remedies to treat ailments due to their easy availability and lower cost compared to synthetic compounds [3,4]. They also demonstrate a number of promising activities against various health problems (e.g., respiratory and gastrointestinal disorders) and show anti-inflammatory, spasmolytic, antioxidant, sedative, antimicrobial, antiviral, antiseptic, anti-diabetic, immunostimulant, and hepatoprotective activities [5,6,7]. In addition, numerous phytoconstituents and plenty of chemical compounds with different biological and pharmacological activities have been isolated and identified from medicinal plants [8,9,10]. For instance, Batiha et al. [8], as well as Beshbishy et al. [9], reported the antiprotozoal activity of chalcones and ellagic acid, the naturally derived phytoconstituents isolated from herbal extracts against *Plasmodium*, *Leishmania*, *Trypanosoma*, *Babesia,* and *Theileria* parasites. These phytochemical compounds have been shown to be lead compounds for the development of new synthetic compounds, with higher efficacy and lower toxic side effects [11].

**Figure 1:**
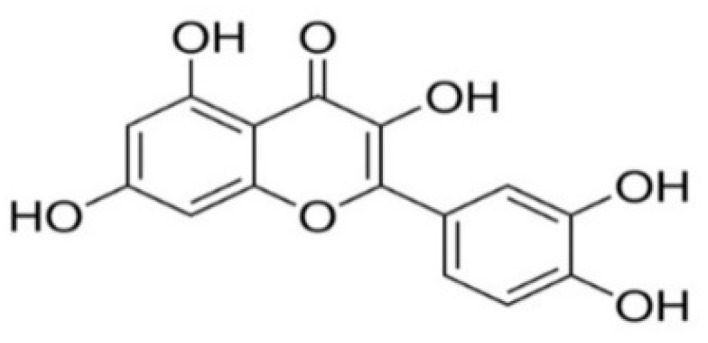
Quercetin’s chemical structure.

Quercetin (Figure 1: (2-(3,4-dihydroxyphenyl)-3,5,7-trihydroxy-4-Hchromen-4-one)) is classified as a flavonol, which is one of the six subcategories of flavonoid compounds and is the major polyphenolic flavonoid found in various vegetables and fruits, such as berries, lovage, capers, cilantro, dill, apples, and onions [12]. It is yellow in color and completely soluble in lipids and alcohol, insoluble in cold water, while sparingly soluble in hot water. Quercetin’s name derives from the Latin word “*Quercetum*”, which means Oak Forest, and also belongs to the flavonol category, which is not produced in the human body [13]. The name of the International Union of Pure and Applied Chemistry (IUPAC) and the chemical formula of quercitin are as follows: 2-(3,4-dihydroxyphenyl)-3,5,7-trihydroxychromen-4-one and C_15_H_10_O_7_, respectively. Quercetin is one of the most important plant molecules that has shown many pharmacological activities, such as being anticancer, antiviral, and treating allergic, metabolic, and inflammatory disorders, eye and cardiovascular diseases, and arthritis [14]. It has also shown a wide range of anticancer properties, and several reports indicate its efficacy as a cancer-preventing agent. Quercetin also has psychostimulant properties and has been documented to prevent platelet aggregation, capillary permeability, and lipid peroxidation, and enhance mitochondrial biogenesis [15]. The current review aims to further understand quercetin’s beneficial and pharmacological effects, as well as its clinical application and concerns around safety.

## 2. Bioavailability and Pharmacokinetics of Quercetin

Previous animal and human research have reported poor oral bioavailability of quercetin after a single oral dose due to macronutrient absorption [16]. For instance, quercetin is ingested in the form of glycosides, and glycosyl groups are released during chewing, digestion, and absorption. Afterward, quercetin glycosides are converted into aglycone in the intestine before they are absorbed into enterocytes by the action of β-glycosidases enzymes. According to Walle et al. [17], previous studies have reported that intestinal and oral bacteria are involved in this enzymatic hydrolysis. Quercetin is a lipophilic compound, so it is assumed that it can cross the intestinal membranes by simple diffusion, and theoretically, this absorption is better than its glycoside forms which reach the intestines without degradation [18]. To date, a number of human studies have been conducted on the bioavailability of quercetin glycosides extracted from different species. For example, quercetin glycosides from onions were absorbed in patients with ileostomy at a higher percentage than pure aglycone, which has been reported by Hollman et al. [19]. On the other hand, Scholz and Williamson [20] documented the existence of significant amounts of aglycone in ileostomy fluid taken from patients with ileostomy who had eaten a meal with onions. They also reported the presence of a high quantity of quercetin glycosides and a small amount of quercetin aglycone, but quercetin glycosides were not observed in the fluid. One possible explanation is that the hydrolysis of quercetin glycosides takes place as it is converted by β-glycosidases enzymes to aglycone. These enzymes are found in the small intestine, and most of them are then absorbed. Ferry et al. [21] studied the pharmacokinetic properties of intravenous quercetin injection in cancer patients at dose levels of 60–2000 mg/m^2^. They determined that 945 mg/m^2^ was a safe dose of quercetin, while its toxic dose was reported to cause emesis, hypertension, nephrotoxicity, and decreased serum potassium. The distribution and elimination half-life of intravenous quercetin were found to be 0.7–7.8 min and 3.8–86 min, respectively, whereas its clearance and distribution volume were 0.23–0.84 L/min/m^2^ and 3.7 L/m^2^, respectively. Erlund et al. [22] examined the pharmacokinetic properties of 8, 20, and 500 mg quercetin aglycone orally in healthy participants. Graefe et al. [23] also studied the pharmacokinetic properties of quercetin and maintained a dose level of up to 200 mg and demonstrated that quercetin C_max_ and T_max_ were 2.3 ± 1.5 µg/mL and 0.7 ± 0.3 h, respectively.

## 3. Sources of Quercetin and Its Pharmacological Activity

**Figure 2:**
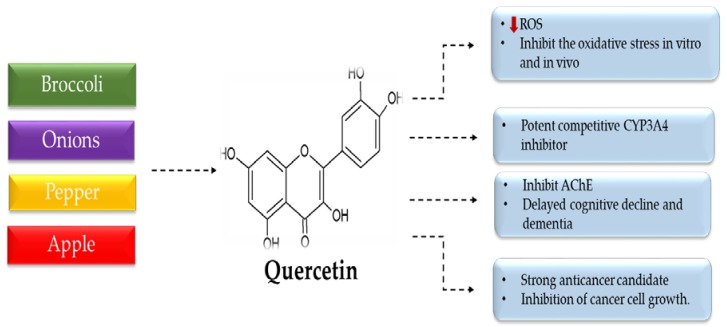
The pharmacological activity of quercetin.

Quercetin is one of the most significant bioflavonoid compounds found in vegetables, grains, and fruits for more than 20 plant species—namely, *Foeniculum vulgare*, *Curcuma domestica valeton*, *Santalum album*, *Cuscuta reflexa*, *Withania somnifera*, *Emblica officinalis*, *Mangifera indica*, *Daucus carota*, *Momordica charantia*, *Ocimum sanctum*, *Psoralea corylifolia*, *Swertia chirayita*, *Solanum nigrum*, and *Glycyrrhiza glabra, Morua alba*, *Camellia sinensis* [3], *Allium fistulosum*, *A. cepa*, *Calamus scipionum*, *Moringa oleifera*, *Centella asiatica*, *Hypericum hircinum*, *H. perforatum*, *Apium graveolens*, *Brassica oleracea* var. italica, *B. oleracea* var. sabellica, *Coriandrum sativum*, *Lactuca sativa*, *Nasturtium officinale*, *Asparagus officinalis*, *Capparis spinosa*, *Prunus domestica*, *P. avium*, *Malus domestica*, *Vaccinium oxycoccus*, and *Solanum Lycopersicum* [12]. It pharmacologically possesses antiobesity, anti-inflammatory, and vasodilator effects, and antioxidant, immunostimulant, anti-diabetic, antihypertensive, antiatherosclerosis, and antihypercholesterolemic activities (Figure 2) [24]. It is available as a food supplement in capsule and powder form.

### 3.1. General Pharmacological/Biochemical Properties of Quercetin 

Some of the sources and pharmacological activity of quercetin are shown below in Table 1.

### 3.2. Antioxidant Activity

Interestingly, the beneficial effects of quercetin have been attributed to its antioxidant activity. Quercetin is a large class of flavonoids, consisting of five classes of hydroxyl groups, 3,5,7,3’, and 4’ of the basic flavonol skeleton. Some of these classes of hydroxyls are glycosylated to different quercetin glycosides and form the major quercetin derivatives. It is noteworthy that several studies have shown the relationship between the structural activities of quercetin and its derivatives on antioxidant and anti-inflammatory activities [25]. They found that the modification of quercetin reduces its antioxidant activity, and the total activity was found to be as follows: quercetin > tamarixetin = isorhamnetin > quercetin-3-O-glucuronide > isorhamnetin-3-O-glucoside > quercetin-3,5,7,3’,4’-pentamethylether > quercetin-3,4’-di-glucoside, indicating that the 3-hydroxyl quercetin group plays a significant role in antioxidant activity [26]. Moreover, Lesjak et al. [25] reported that methylated quercetin metabolites (e.g., isorhamnetin and tamarixetin) showed higher antioxidant activity than quercetin by inhibiting lipid peroxidation. The antioxidant activity of quercetin has been documented because it can scavenge reactive oxygen species [27]. Quercetin is used to prevent cancer by modulating oxidative stress factors and antioxidant enzymes to prevent the spread of various cancers, such as lung, prostate, liver, breast, colon, and cervical cancers. The in vivo study examined the antioxidant activity of quercetin compared to carcinogen and testosterone by measuring histology and oxidative stress markers, such as reduced glutathione (GSH), lipid peroxidation (LPO), and hydrogen peroxide (H_2_O_2_) in rats. They found that rats treated with carcinogen and testosterone had higher levels of LPO and H_2_O_2_ and lower levels of GSH compared to quercetin-treated rats [28]. Sharmila et al. [29] reported that quercetin increased the levels of apoptosis proteins and antioxidant enzymes in animals infected with prostate cancer. Moreover, they documented that quercetin regulated the expression of androgen receptors (AR), protein kinase B (AKT), insulin-like growth factor receptor 1 (IGFIR), and cell proliferation and anti-apoptotic proteins that are increased in cancer. In addition to that, quercetin has been documented to lower malondialdehyde (MDA) content while increasing catalase and superoxide dismutase (SOD) activity to control the anti-inflammatory and anti-apoptosis processes to effectively protect the heart from secondary cardiac dysfunction due to oxidative stress and inflammation [30]. Quercetin also reduces the overproduction of ROS, damage caused by trauma, improves TNF-α, and prevents myocardial cell injury caused by Ca^2+^ overload. Quercetin can thus effectively prevent injury caused by oxidative stress [31]. 

Interestingly, the antioxidant efficacy of quercetin has been documented in earlier reports to reduce and inhibit oxidative stress and damage, both in vivo and in vitro [32,33]. For illustration, Moretti et al. [34] demonstrated the efficacy of quercetin in the prevention of lipid peroxidation caused by *tert*-Butyl hydroperoxide in human sperm cells in vivo. An additional report in rats revealed that quercetin administered at dose levels of 25–50 mg/kg showed antioxidant action against oxidative stress, which results in streptozotocin-induced diabetes mellitus [35]. Moreover, it has been reported that quercetin acts as a stabilizer in the polyethylene when it is administered at a dose level of 250 µg/mL in addition to its antioxidant activity, and therefore the polymer’s residual stability is increased for a long time [36]. Furthermore, the use of quercetin as a chelating agent in chelation therapy for the removal of toxic metallic ions such as cadmium as quercetin-cadmium complexes has been shown to have a high stability constant (Kf) value [37]. Quercetin reduces oxidative stress by controlling the oxidant–antioxidant balance. Several studies have reported that quercetin inhibits oxidative damage caused by acrylamide, brain damage caused by radiation in rats, neurodegenerative disorders, oxidative stress induced by cadmium fluoride, and nerve damage in diabetic rats’ retinas. Quercetin protects the nerves, brain, or other body cells from oxidation-induced damage by regulating the antioxidant levels [31]. Quercetin prevents free radicals and enhances the body’s antioxidant defense systems and therefore reduces oxidative stress, including the production of nicotine-induced ROS for the treatment of diseases such as nicotine addiction [38]. In vivo studies have shown that quercetin has antioxidant and hepatoprotective activity against acute hepatic injury caused by tertiary butyl hydrogen peroxide. Quercetin effectively protects cells from genetic toxicity and radiation-induced damage by scavenging free radicals and increasing the levels of endogenous antioxidants [39].

### 3.3. Antiviral Activity

Quercetin has shown antiviral activity towards a wide range of viruses. For instance, quercetin has been documented for its efficacy against the human T-lymphotropic virus 1, as well as the Japanese encephalitis virus (JEV) caused by Japanese encephalitis, the mosquito-borne disease [40,41]. Furthermore, quercetin has been reported to suppress the dengue virus type-2 and hepatitis C virus by suppressing the nonstructural protein 3 protease activity [42,43]. Other Quercetin formulations, such as quercetin-3-O-β-D-glucuronide, quercetin-enriched lecithin formulations, and quercetin 7-rhamnoside have been reported for their efficacy against the porcine epidemic diarrhea virus and influenza-A virus, respectively [44,45,46].

### 3.4. Antimicrobial Activity 

Quercetin has exhibited potent bacteriostatic activity against different strains of bacteria, such as *Salmonella enterica* serotype Typhimurium, *Pseudomonas aeruginosa*, *P. fluorescens*, *Helicobacter pylori*, *Staphylococcus epidermidis, S. aureus*, *Yersinia enterocolitica*, *Micrococcus luteus, Campylobacter jejuni,* and *Escherichia coli,* which have been more effective against Gram-positive than Gram-negative bacteria [47]. Jaisinghani. [48] also reported its efficacy against *Shigella flexeneri* NCIM5265 and *Lactobacillus casei var Shirota.* Strikingly, Osonga et al. [49] documented that quercetin derivatives (e.g., quercetin 4′,5-diphosphate (QDP), quercetin 3′,4′,3,5,7-pentaphosphate (QPP), quercetin 5′-sulfonic acid (QSA)) resulted in highly biocompatible, soluble, and potent antibacterial activity with 100% inhibition of *Listeria monocytogenes*, *Pseudomonas aeruginosa*, and *Aeromonas hydrophila*. Moreover, quercetin revealed the strongest antifungal activities against *Candida albicans, Cryptococcus neoformans,* and *Aspergillus niger* [50].

### 3.5. Antiprotozoal Activity

Several reports have demonstrated the growth inhibitory effects of quercetin against various protozoan parasites, namely *Toxoplasma*, *Babesia*, *Theileria*, *Trypanosoma,* and *Leishmania*. Interestingly, quercetin is well-known for its growth inhibitory efficacy against *Trypanosoma brucei rhodesiense, T. brucei brucei, T. cruzi,* and *Leishmania donovani* parasites in vitro and in vivo [51]. It resulted in potent leishmanicidal and trypanocidal activity in vitro, with an IC_50_ of 1.0 μg/mL and 8.3 μg/mL, respectively, while in an in vivo experiment, among six tested flavonoids, only quercetin showed in vivo activity by inhibiting the multiplication of *L. donovani.* Moreover, Weiss et al. [52] documented the remarkable inhibitory effects of quercetin against *Toxoplasma gondii* by preventing the heat shock protein 90 (hsp90), hsp70, and hsp27 synthesis, and thus suppressing the induction of bradyzoite development. Lehane and Saliba. [53] described the antiplasmodial activity of quercetin against a chloroquine-sensitive (3D7) and chloroquine-resistant (7G8) strain of *Plasmodium falciparum.*

### 3.6. Anti-Inflammatory Effects of Quercetin 

Quercetin has been shown to be a long-lasting anti-inflammatory agent with good anti-inflammatory activity. Several in vitro studies have shown that quercetin prevents the development of lipopolysaccharide (LPS)-mediated tumor necrosis factor-α (TNF-α) in macrophages and the development of IL-8 induced LPS in lung A549 cells [54]. In addition, quercetin can inhibit TNF-α and Interleukin (IL)-1α levels of LPS-induced mRNA, which results in reduced apoptotic neuronal cell death caused by microglial activation [55]. Quercetin suppresses the production of inflammatory enzymes (e.g., lipoxygenase (LOX) and cyclooxygenase (COX)). It regulates inflammation caused by LPS by inhibiting Src- and Syk-mediated phosphatidylinositol-3-Kinase (PI3K)-(p85) tyrosine phosphorylation and subsequent complex formation of Toll-like Receptor 4 (TLR4)/MyD88/PI3 K, which restricts downstream signaling pathway activation in RAW 264.7 cells [56]. It may also inhibit the release of pro-inflammatory cytokines, tryptase, and histamine from human umbilical cord blood-derived mast cells; this inhibition is likely to involve the inhibition of calcium influx and Phospho-protein kinase C (PKC) [54]. Quercetin substantially stimulates the gene expression and the development of interferon-γ (IFN-γ) derived from T helper cell-1 (Th-1) and down-regulates IL-4 derived from Th-2 by normal peripheral blood mononuclear cells (PBMC). Quercetin is also known to have inhibitory activity against COX-2, nuclear factor-kappa B (NF-*κ*B), activator protein 1 (AP-1), mitogen-activated protein kinase (MAPK), reactive nitric oxide synthase, (NOS) and reactive C-protein (CRP) expression that causes inflammation [57]. Due to its weak absorption through the surface of the skin, quercetin and its glycoside derivatives have been reported to be ineffective against topical inflammation, while pentamethyl ether, which is a quercetin derivative, has shown potent anti-inflammatory activity with higher absorption through the skin’s surface in the rat [58]. Several reports have been documented that quercetin prevents the secretion of iNOS, IL-1β, and TNF-α caused by bacterial LPS in macrophages, TNF-α secretion in RAW2647 cells, and cytokine-stimulated vascular cell adhesion molecules (VCAM-1) and intracellular cell adhesion molecule (ICAM-1) expression, and E-selection in human umbilical vein endothelial cells. Notably, quercetin and its glycoside rutin have shown a reduction in the inflammatory markers TNF-α and IL-6 in NASH mice [59].

### 3.7. Efficacy in Diseases

#### 3.7.1. Anticancer Activity of Quercetin

Quercetin has been documented to possess anticancer activity both in vitro and in vivo. In in vitro experiments, the anticancer efficacy of quercetin against different cell lines was determined by the prevention of angiogenesis in tamoxifen-resistant cancer, while its in vivo efficacy was attributed to its antioxidant activity [60,61,62]. According to Gibellini et al. [63], quercetin is considered to be a strong anticancer candidate due to its chemoprotective activity through metastasis and apoptosis against tumor cell lines. Moreover, Du et al. [64] demonstrated the potent efficacy of the quercetin-doxorubicin combined treatment in persistent T-cell tumor-specific responses, resulting in improved the immune responses against breast tumor growth [64]. It is worth noting that quercetin prevents the proliferation of several types of cancers (e.g., breast, lung, prostate, cervical, liver, and colon cancer) and it acts by a various mechanism of actions, including cellular signaling, binding to cellular receptors and proteins, and inhibiting enzymes responsible for carcinogen activation [65]. Recently, quercetin has been reported to increase the chemosensitivity of breast cancer cells to doxorubicin by preventing cell propagation and invasion that promote cell apoptosis. Furthermore, quercetin demonstrated an inhibitory effect on MCF-7 and MDA-MB-231 human breast cancer cell lines by regulating miR-146a expression, cell apoptosis induction, Caspase 3 activation, and mitochondrial pathways [66]. Quercetin also exhibits anti-colon cancer effects with the TLR4- and NF-κB-mediated signaling pathway, and it was found that quercetin showed significant inhibition of human colon cancer proliferation in CACO-2 and SW-620 cells by preventing the NF-κB pathway, down-regulation of B-cell lymphoma 2, and up-regulation of Bcl-2-associated X protein [67].

#### 3.7.2. Quercitin Hepatoprotective and Antihypertensive Activities 

Recently, an in vivo study found that quercetin increased heme oxygenase 1 activity in D-galactosamine- and LPS-treated rats by lowering plasma concentrations of alanine aminotransferase and stimulating its hepatotoxic and hepatoprotective activity [68]. Moreover, Liu et al. [69] revealed the ability of quercetin to treat ethanol-induced oxidative damage in rat hepatocytes, suggesting that quercetin may be an appropriate hepatoprotective natural product. Duarte et al. [70] reported that quercetin had antihypertensive activity in spontaneously hypertensive rats, and noted that quercetin had induced a dose-dependent, advanced, and potential reduction in pressure of the blood when given chronically to several hypertensive rat models.

#### 3.7.3. The Important Role of Quercetin in the Treatment of Alzheimer’s Disease 

Alzheimer’s disease (AD) is considered to be the most prevalent cause of dementia, a chronic neurodegenerative disorder characterized by memory loss and mental deficits, such as apraxia, aphasia, and agnosia, and is associated with neuroinflammatory processes in the central nervous system [71,72]. The memory contains several types: visual, olfactory, episodic, and vocal. These are classified into two categories: explicit (active or passive recall of facts) and implicit (nonverbal habitual memory) [73]. 

Oxidative stress is caused by a free radical imbalance in the body and is included in the establishment of neurodegenerative disorders involving AD. Flavonoids like quercetin have different activities in the vascular system, leading to several modifications in cerebrovascular blood flow, which can alter the neuronal morphology that causes neurogenesis and angiogenesis. In addition to that, it can also protect neurons from neurotoxin-induced injury. Rich food consumption of flavonoids limits neurodegeneration and inverts the age-related injury to cognitive performance [74]. Moreover, quercetin and ascorbic acid combined treatment have been shown to reduce the prevalence of oxidative injury to human lymphocytes and neurovascular structures in the skin and thus prevent neuron injury, which particularly protects the brain cells from oxidative stress that leads to AD and other neurological conditions [13]. 

Quercetin’s beneficial effects against AD are ascribed due to its inhibitory efficacy against acetylcholinesterase (AChE) [72]. Recently, in vivo experiments have documented the ability of quercetin to reduce the oxidative stress caused by 6-hydroxydopamine in the neurons of rats [75]. Another study conducted on healthy P19 neurons revealed that neuron survival is not affected by quercetin, while it depletes the glutathione content that may affect the functioning of the nervous system [76]. Furthermore, recent findings have shown that quercetin improves the pathology of AD and related cognitive deficits in triple-transgenic, aged AD mice [77]. Additionally, combined oral ingestion of quercetin with fish oil improved neuroprotection in 3-nitropropionic acid-treated rats or chronic rotenone-treated rats [78,79]. 

In the AD, quercetin acts by the following mechanism of action: α-tocopherol (vitamin E), a type of antioxidant that enhances quercetin penetration through the blood–brain barrier (BBB), which leads to significant improvement in quercetin concentration and thus reduces the prevalence of oxidative damage in the brain. Moreover, quercetin acts by activating the NF-E2-related factor 2- antioxidant responsive element (Nrf2-ARE) that offers a neuroprotective effect against oxidative injury and cell death. Recently, previous studies have shown that the formation and deterioration of undisciplined protein aggregates in various neurodegenerative diseases, such as Huntington’s diseases, Alzheimer’s, Parkinson’s, and amyotrophic lateral sclerosis may be altered by the Nrf2-ARE pathway [80,81].

## 4. Combination Therapy of Quercetin with Other Drugs 

The combined effect of quercetin with other antioxidants (e.g., ascorbic acid), decreases the prevalence of oxidative damage in human lymphocytes and neurovascular structures in the skin and inhibits the neuron injury. Moreover, it has been reported to possess a potent effect against AD by protecting the brain cells from oxidative stress that induces tissue damage, resulting in AD and other neurological conditions [13]. Notably, quercetin has been documented to possess neuroprotective and neurotoxic activity, and its combined effect with fish oil has shown neuroprotective efficacy in rat brains and has subsequently shown beneficial effects against neurodegenerative diseases [79]. Quercetin is well-known to influence the pharmacokinetics of different drugs, such as curcumin and resveratrol by controlling their transfer and metabolism, as well as some of its significant activities, including CYP3A4, P-gp efflux pump, and phenol sulfotransferase (SULT 1A1) inhibition. These combined treatments have resulted in an increase in curcumin and resveratrol permeability and acute bioavailability compared to single treatments [82]. Moreover, Sahyon et al. [83] investigated the combination effect of sulfamethoxazole with quercetin against *S. aureus*, and quercetin has been shown to reduce the side effects of sulfamethoxazole while improving its bactericidal efficacy, indicating the importance of this combination therapy for the treatment of human clinical cases. Also, Qu et al. [84] revealed the synergetic effect of quercetin-tetracycline combination treatment against multi-drug resistant (MDR) *E. coli* by disrupting the bacterial cell envelope, thus improving its permeability and cell lysis. Quercetin has been documented to improve the antifungal efficacy of amphotericin B against *Candida* sp and *Cryptococcus neoformans* strains by reducing its toxic effect [85]. Another study demonstrated the potent synergistic efficacy of quercetin against fluconazole-resistant strains of *Candida tropicalis* by enhancing mitochondrial membrane alterations that affect the mitochondrial respiratory function and inhibiting rhodamine-123 accumulation in the mitochondria [86]. Recently, quercetin has been documented to possess synergistic effects when combined with chemotherapeutic drugs (e.g., cisplatin) [65].

## 5. Dose Use 

Typical dietary quercetin intake based on fruit and vegetable consumption is estimated to range from 5 to 100 mg per day. Heavy consumption of foods rich in quercetin, such as apples or onions, could lead to a daily intake of up to 500 mg [87,88,89]. The effective dose is increased when taken with a fatty meal or in the presence of apple pectin, oligosaccharides, and lecithin [87,90]. Most clinical studies use quercetin at 500 to 1000 mg per day in divided doses [91,92]. As a supplementary food, 2 weeks of quercetin 50 mg achieved a 178% increase in serum levels, while quercetin 100 mg had a 359% increase in the serum levels, and quercetin 500 mg had a 570% increase in the serum levels, although with wide individual variation [93]. Based on animal studies, quercetin accumulates in the lungs, liver, kidneys, and small intestines, with lower levels seen in the brain, heart, and spleen. It is eliminated through the renal, fecal, and respiratory systems [89,94].

## 6. Metabolism and Excretion of Quercetin

After quercetin administration and absorption, it is transferred to the liver where the first and second phases of metabolism take place, resulting in metabolic products entering the bloodstream for distribution in the body’s tissues [95]. Mullen et al. [96] examined the main metabolites of quercetin in the urine and plasma of healthy people after the ingestion of onions. Three major metabolites were identified in the plasma—namely, quercetin-30-sulfate, quercetin-3-glucuronide, and quercetin-3-sulfate with the highest concentrations at 0.8 and 0.6 h, while quercetin-30-glucuronide, quercetin-diglucuronide, isorhamnetin–glucuronide sulfate, isorhamnetin-methyl quercetin, and diglucuronide isorhamnetin-glucuronide were the major urinary metabolites that reached their highest concentrations after 4 h. Notably, quercetin had a short half-life and rapid clearance in the blood, and its metabolites appeared in the plasma 30 min after ingestion, but considerable amounts were excreted over 24 h [97]. Moon et al. [94] identified the aggregation of quercetin conjugates in human plasma following multiple administrations of quercetin-rich foods. The highest concentration of quercetin metabolites was identified following the uptake of onions, and sulfate and glucuronide metabolites were significantly (*p* < 0.05) elevated from 0.04 to 0.63 µM in the plasma of fasting participants. 

**Figure 3:**
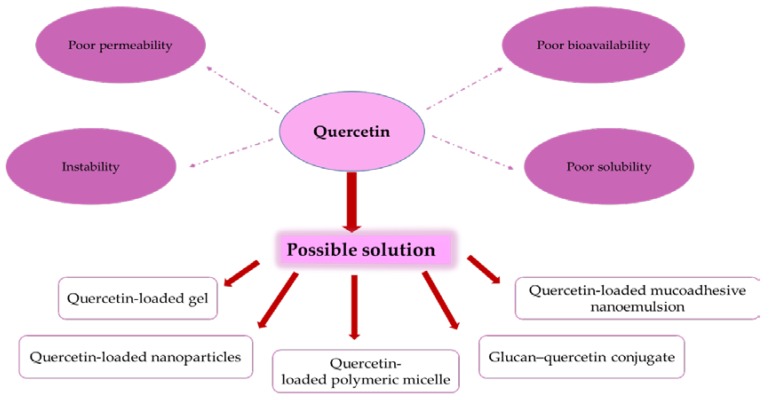
Quercetin formulations for improving its bioavailability.

The use of quercetin in the pharmaceutical industry is limited due to its poor bioavailability, poor aqueous solubility, poor permeability, and instability. Therefore, several studies have been conducted to modify its structure to increase its water solubility and bioavailability and thereby enhance its antioxidant and antimicrobial activity [98]. Recently, new quercetin preparations have appeared, including quercetin-loaded gel, quercetin-loaded mucoadhesive nanoemulsion, quercetin-loaded nanoparticles, and quercetin-loaded polymeric micelle, which may provide new drug formulations for research and development (Figure 3) [31]. Moreover, quercetin bioavailability has also been improved by structural modification with glucoside–sulfate conjugates and the preparation of some complex ionic complexes, such as quercetin–germanium nanoparticles, calcium phosphate–quercetin nanocomplex (CPQN), and glucan–quercetin conjugate that showed higher antioxidant activity than free quercetin [99]. Quercetin also exhibits excellent antioxidant activity and scavenging capacity when combined with metal ions, such as cadmium, vanadium, calcium, magnesium, copper, cobalt, iron, and ruthenium [31]. 

## 7. Toxic Side Effects of Quercetin

Quercetin is known to be a mutagenic agent based on the Ames test; however, most in vivo animal studies have shown that quercetin is a safe compound without any carcinogenic effects. It is worth noting that in 1999, the International Agency for Research on Cancer (IARC) stated that quercetin should not be listed as a human carcinogen compound [100,101]. There is no definite proof of quercetin teratogenic activity on embryonic growth; however, in vitro studies suggest that quercetin can have a mild negative impact on fetal growth and demonstrate protective efficacy against toxic agents [102]. In vivo experiments have shown that quercetin resulted in a small increase in the prevalence of malignant tumors to the young offspring of mice lacking DNA repair [103]. An in vivo experiment performed on a four-week rat showed that the ratio of liver and kidney weights increased remarkably in rats fed greater than 314 mg and 157 mg quercetin/kg body weight/day, respectively. Moreover, a pro-oxidant efficacy was observed at doses higher than 157 mg quercetin/kg body weight/day [104]. Quercetin was usually well-tolerated in human clinical studies. Notably, quercetin administration for several months at a concentration higher than 1000 mg/day did not show any side effects on serum electrolytes, kidney, and liver function blood parameters, or hematology. At present, co-administration of high quercetin doses with digoxin is known to be the greatest cause of toxicity; thus, the use of quercetin in digoxin-treated patients should be restricted before more information on appropriate dosage levels is available [105]. Quercetin shows mutagenicity in vitro in the Ames test, and reports of mutagenicity in the 1970s have led to concerns about its safety [87]. Under certain circumstances, quercetin exhibits both radical scavenging and pro-oxidant activity [88]. 

The majority of in vivo experiments have shown that quercetin is not a carcinogen and may be protective against Geno toxicants. Dietary quercetin, faced with the first-pass metabolism in the intestine and liver, is almost completely metabolized, reducing the potential for toxicity. At oral supplemental doses higher than 1000 mg per day taken for up to three months, no evidence of toxicity has been found; however, data on long-term safety at high doses are lacking [87]. Nephrotoxicity has been reported with the use of high-dose IV quercetin in patients with compromised health [89]. 

## 8. Quercetin-Drug Interaction

Quercetin has been reported to competitively bind to bacterial DNA gyrase and is, therefore, contraindicated to be administered with fluoroquinolone antibiotics [106]. Moreover, quercetin is a potent competitive inhibitor of CYP3A4 (the enzyme responsible for drug degradation in the body) and was thus predicted to increase the serum concentrations of drugs (e.g., diltiazem) that are metabolized by this enzyme [107]. Therefore, research towards the optimum mechanism of action of natural compounds that prevents the adverse effects of plants and the development of new molecules with new pharmacological effects will continue [108,109,110].

## 9. Conclusions

This review examined the therapeutic and toxic activities of quercetin. Quercetin is the major polyphenolic flavonoid present in several food products that have shown many pharmacological activities, such as anticancer, antiviral, antiprotozoal, and antimicrobial effects, treatment of allergic, metabolic, and inflammatory disorders, eye and cardiovascular diseases, and arthritis. Previous studies documented the poor oral bioavailability of quercetin after a single oral dose, as its absorption was impaired by the macronutrients. It is a famous AChE inhibitor and has been used in treating neurodegenerative diseases, including AD. Quercetin has been documented to have both neurotoxic and neuroprotective activities, and its combined effect with fish oil and ascorbic acid has demonstrated beneficial effects against neurodegenerative diseases. The finding that quercetin can combine with other drugs is a property that can be explored in the chemotherapies’ development against AD. However, due to the presence of adverse side effects, its therapeutic use as a treatment has been banned. Moreover, quercetin has been confirmed to be competitively bound to bacterial DNA gyrase and is, therefore, contraindicated to be administered with fluoroquinolone antibiotics.

## Figures and Tables

**Table 1:** Sources of quercetin and its traditional uses.

Plant Name	Family	Pharmacological Activity
*Apium graveolens*	Apiaceae	Lowers blood pressure and glucose, anti-inflammatory, antibacterial
*Allium fistulosum*	Amaryllidaceae	Spring onions as food ingredients
*Allium cepa* (red onions)	Amaryllidaceae	Immunostimulant, cardioprotective, antioxidant
*Calamus scipionum*	Arecaceae	Source of cane
*Moringa oleifera*	Moringaceae	Multipurpose medicinal use anti-inflammatory, antihypertensive, antibacterial
*Centella asiatica*	Apiaceae	Wound healing
*Hypericum hircinum*	Hypericaceae	Antioxidant
*Hypericum perforatum*	Hypericaceae	Major depressive disorders, Neurological effects
*Brassica oleracea* var. sabellica (Kale)	Brassicaceae	Reduce the risk of stroke, reduces blood glucose, neuropathy
*Brassica oleracea* var. italica (broccoli)	Brassicaceae	Edible plant prevents fluid retention and cancer
*Solanum lycopersicum*	Solanaceae	Food supplement and salads
*Coriandrum Sativum*	Apiaceae	Reduce blood pressure, cholesterol, and dyspepsia
*Morua alba*	Moraceae	Diet
*Nasturtium officinale*	Brassicaceae	Reduces the risk of cancers
*Asparagus officinalis*	Asparagaceae	Antineoplastic, antiulcer, antitussive
*Lactuca sativa*	Asteraceae	Iron deficiency anemia, osteoporosis
*Prunus domestica*	Rosaceae	Laxative
*Malus domestica*	Rosaceae	Decrease the risk of cardiovascular disease and cancer
*Capparis spinosa*	Capparaceae	Vermifuges, disinfectants, antiatherosclerotic agent
*Vaccinium oxycoccus*	Ericaceae	Urinary tract infections
*Prunus avium*	Rosaceae	Tonic, astringent, diuretic
*Camellia sinensis*	Theaceae	Antiviral, antispasmodic, analgesic, antidiabetic, bronchodilator

## Abbreviations

AD	Alzheimer’s disease
IUPAC	International Union of Pure and Applied Chemistry
Kf	stability constant value
JEV	Japanese encephalitis virus
QDP	quercetin 4′,5-diphosphate
QPP	quercetin 3′,4′,3,5,7-pentaphosphate
QSA	quercetin 5′-sulfonic acid
hsp90	heat shock protein 90
COX	cyclooxygenase
LOX	lipoxygenase
TLR4	Toll-like Receptor 4
PKC	Phospho-protein kinase C
Th-1	T helper cell-1
NF-*κ*B	nuclear factor-kappa B
AP-1	activator protein 1
MAPK	mitogen-activated protein kinase
NOS	nitric oxide synthase
CRP	reactive C-protein
IL-1β	interleukin-1β
TNF-α	tumor necrosis factor-α
LPS	lipopolysaccharide
VCAM-1	vascular cell adhesion molecules
ICAM-1	intracellular cell adhesion molecules
CPQN	calcium phosphate–quercetin nanocomplex
BBB	blood-brain barrier
Nrf2-ARE	NF-E2-related factor 2-antioxidant responsive element
SULT 1A1	phenol sulfotransferase
MDR	multi-drug resistant
IARC	International Agency for Research on Cancer
AChE	acetylcholinesterase
